# Evaluation of an Integrated Intensive Cognitive Behavioral Therapy Treatment Within Addiction Care

**DOI:** 10.1007/s11414-019-09657-5

**Published:** 2019-04-17

**Authors:** Kourosh Bador, Nóra Kerekes

**Affiliations:** 1 AGERA KBT AB, Gothenburg, Sweden; 2 Department of Health Sciences, University West, 46186 Trollhättan, Sweden

## Abstract

The study aimed to evaluate an integrated intensive cognitive behavioral therapy (CBT) group treatment for people with substance-related syndrome in outpatient care and to identify eventual gender differences. The study population consisted of 35 outpatients (18 male, 17 female) at a clinic in Western Sweden. The patients completed a four-month period of intensive group therapy and participated in the data collection at admission and discharge. The data were collected using the following inventories: Beck Depression and Anxiety Inventories, Rosenberg Self-Esteem Scale, Hopelessness Scale, and Trait Hope Scale. Results showed decreases in anxiety, depression and experience of hopelessness, and increases in self-esteem and hope. In females, the most dramatic improvement was measured for the anxiety and depression attributes, while in males the strongest effect was measured for hope and self-esteem. This study provides clinical evidence of the positive effects of integrated intensive CBT in outpatient care of people with substance-related syndrome.

## Introduction

According to The Public Health Agency of Sweden,^1^ about 23% of men and 12% of women in the Swedish population use illicit drugs at some time in their lives, while 2% of men and less than 1% of women state that they have used some kind of illicit drug in the past 30 days. In 2011, there were approximately 500,000 people suffering from substance use and dependency in Sweden.^2^ The Swedish findings about gender differences in risk factors and consequences of drug addiction are similar to those found in other countries.^3^ Twice as many women as men use classified narcotics or addictive medicines without physician prescription or at higher doses than prescribed.^1^ When women initiate substance use, they tend to increase their consumption of drugs more quickly^4^ and develop dependency more rapidly^5, 6^ than men. These facts reflect male-female differences in the possibilities of abusing pharmaceuticals on the basis of the impact of the social or cultural environment.^7^ They also suggest variation among men and women in the biological response and problem progression in relationship to substance use and dependence.^8^

Women with frequent drug use are substantially more vulnerable than men. They also have less social support and greater co-existing mental illness. It is less likely that women seek help for drug problems than men because women are more prone to overcoming the challenges that limit their access to drugs than to seek help for their addiction.^9^ Globally, one out of three persons with substance-related syndrome is a woman; yet only one out of five patients in drug treatment is a woman. Men often seek help at the behest of their family, employer, or the criminal justice system. Substance use and dependence in women is often associated with other problems such as personality syndrom diagnoses or prostitution which do not necessarily entail contact with the healthcare system.^8^

That people with substance-related syndrome have multi-dimensional problems, including coexisting mental illness and social problems, is more frequently a rule than an exception.^10^ Nevertheless, even in highly developed social support systems, such as in Sweden, only about 20% of people with substance-related syndrome are known to the healthcare system and social services.^2^

Traditionally, the most common approach for treating individuals with addiction and co-occurring psychiatric disorders is to identify one disorder as “primary” and the other(s) as “secondary.” In this type of sequential treatment strategy, one focuses on treating the “primary disorder” first and treats the secondary disorder(s) only when/if the primary disorder is believed to have been fully treated. The challenges in defining the primary diagnosis are obvious and often cause disagreement between clinicians. The issue of the transition between the completion of one treatment and the start of the next can also be a source of concern. Another treatment strategy is the parallel treatment, which means that individuals with coexisting mental illnesses are referred to different facilities or institutions for the simultaneous treatment of addiction and mental illness. It is not uncommon that in this form of approach the patient is expected to coordinate the treatment between different agencies, a responsibility he or she obviously often cannot shoulder satisfactorily.

A new treatment strategy, which has been found to be both efficient and gentler for this patient group, and possibly also more cost-effective, is integrated treatment.^11, 12^ In this approach, there is no need to coordinate between different treatment facilities or agencies and there are no differences between methodologies or ideologies, as the substance use problem and the mental disorder are both treated by the same team implementing an overlapping strategy.

Several randomized trials and naturalistic analyses have compared intensive outpatient treatment with inpatient or institutional care and have found similar results,^13^ such as that intensive outpatient treatment is an effective form of treatment for patients who suffer from substance use and dependence with or without co-occurring mental disorders, and who do not need medical detoxification or round-the-clock care. Intensive outpatient treatment is an important part of the care chain for substance-related care and can offer to promote better health in clients just as effectively as can inpatient or institutional healthcare.^13^

This clinical study aimed to evaluate an integrated intensive cognitive behavioral group treatment for individuals with substance-related syndrome in outpatient care settings and to study eventual gender differences in terms of outcome.

## Method

### Study population

During the study period (October 2014 to September 2017), a total of 50 patients sought outpatient addiction treatment in a cognitive behavioral treatment clinic in Western Sweden. The clinic offers adults with substance-related syndrome a one-year program. During the first four months of the program, the patients receive treatment five days a week. During the remaining eight months of the program, the patients participate in follow-up group sessions once a week. The treatment program is not gender specific; the groups and all treatment moments (excluding individual psychotherapy) include male and female patients at the same time. During the program, the patients work with licensed psychotherapists, behavior scientists, alcohol and drug therapists, and a certified acupuncturist. The clinic’s personnel hold team meetings once a week to secure the progression and quality of the program.

At admission and at discharge, each patient completed a survey comprising several validated instruments (see details under “Instruments”). The completion of the survey took on average of 30 min, during which time the patient was left alone in an undisturbed room.

### Design

The clinic’s outpatient program lasts for one year. The program starts with four months of integrated intensive treatment followed by eight months of follow-up. The treatment is based on cognitive behavioral therapy (CBT). In CBT, the aim is to directly address the patient’s dysfunctional behavior and thinking in order to induce positive emotional changes. The four-month-long integrated intensive treatment includes the following interventions: psychoeducation, cognitive processing, modulation, problem-solving exercises, affect regulation, exposure and response prevention, behavior experiments and activation, mindfulness, skills training, auricular acupuncture (NADA), and home assignments. During this initial four-month period the patient undergoes treatment five days a week. The aim of this study is to evaluate the effects of the first four months of integrated intensive treatment.

At admission, each patient is given information about the clinic’s daily routines and structure, and about the rules and regulations (including ethical considerations) applicable during the treatment period. All patients are given opportunity to ask questions freely before starting the treatment.

### Instruments

The survey began with demographic questions about gender, age, and marital status. These questions were followed by previously validated psychological measures.

The Beck Depression Inventory (BDI)^14^ is a self-assessment scale consisting of 21 questions with a four-point scale to measure depression symptoms and severity in the person during the past seven-day period. The BDI has good internal consistency. The average value for Cronbach’s *α* is 0.86 in psychiatric tests^15^ and in the current study population it was 0.95. It also has adequate validity in comparison with both clinical assessments and other self-assessment instruments.^15^ The Swedish version also has high test-retest reliability (*r* = 0.93).^15^ The previously identified cutoffs were used to define categories as follows: less than or equal to 13 points identified individuals with no or minimum depression; points from 14 to 19 identified individuals with mild depression; from 20 to 28 points identified individuals with moderate depression and 29 points or over (maximum 63) identified individuals with severe depression.^16^

The Beck Anxiety Inventory (BAI)^16^ is a self-assessment scale consisting of 21 questions and measuring the individual’s degree of anxiety by ranks of a Likert scale with the following items: “not at all” (0), “little, has not bothered me a lot” (1), “partly, has been very unpleasant but I could endure it” (2), and “very much, barely could stand it” (3). The BAI’s total score is calculated by adding the scores of the 21 questions. The maximum score is 63. A total score of 0 to 7 points indicates a minimum level of anxiety, from 8 to 15 points indicates a mild experience of anxiety, from 16 to 25 points indicates a moderate experience of anxiety, and 26 points or more indicates a severe experience of anxiety.^16^ The Swedish version of the BAI has an acceptable test-retest reliability (*r* = 0.75).^17^ In this study, the internal reliability of the BAI scale was an acceptable Cronbach’s *α* of 0.93.

The Hopelessness Scale (HS)^18^ intends to measure the degree of experienced hopelessness and pessimism over future expectations, which is an indirect indication for suicide, and also a common sign of depression.^18^ The scale consists of 20 statements to which the test person may answer “true” (1) or “false” (0). The total score is categorized as follows: 0 to 3 indicates no or minimal hopelessness, 4 to 8 indicates a mild experience of hopelessness, 9 to 14 indicates a moderate experience of hopelessness, and 15 to 20 indicates a high experience of hopelessness with definitive risk for suicide.^19^ The reliability of the HS was high (Cronbach’s *α* of 0.86) in this study.

The Rosenberg Self-Esteem Scale (RSES) was developed by Rosenberg^20^ and translated into Swedish by Jonson^21^. The scale contains ten different statements that together measure the participant’s global self-esteem. Of the ten items, five are positive claims and five are negative claims. The answer options are specified on a four-point Likert scale ranging from “fully agree” (3) to “fully reject” (0). Scores from 15 to 25 points indicate normal self-esteem. Scores under 15 points indicate low self-esteem. Scores over 25 indicate high self-esteem.

Tafarodi and Swann^22^ have shown that the RSES measures two distinct but related dimensions, namely “self-competence” and “self-liking”. Self-competence is defined as a person’s experience of being capable. This experience is based on the capability of being successful in achieving goals. Self-liking is defined as a person’s subjective assessment of his or her personal value, not necessarily performance, but according to an internalized set of criteria for social value as moral agents. The reliability of both the total RSES and its self-competence subscale were acceptable (Cronbach’s *α* of 0.81) in this study population, and was close to acceptable for the self-liking subscale (Cronbach’s *α* of 0.63).

The Trait Hope Scale (THS)^23^ is used to measure a person’s experience of hope. The scale measures a global experience of hope and is divided into two subscales, namely “Pathway” (four items) and “Agency” (four items). The hope scale consists of 12 descriptive statements such as “I can think of many ways to get out of a jam” or “I meet the goals that I set for myself”. Of the 12 items, four are distraction questions which are excluded from the actual data analysis. Participants can answer the statements on an eight-point Likert scale ranging from “definitely false” (1) to “definitely true” (8).^24^ The overall THS score can be 64, with the two subscales pathway and agency representing a maximum of 32 points each. In the current study, the overall THS and the agency subscale both had acceptable internal consistency (Cronbach’s *α* of 0.80 and 0.73, respectively), while it was close to acceptable for the pathway subscale (Cronbach’s *α* of 0.65).

### Statistical analyses

All the data was processed using the Statistical Package for the Social Sciences (SPSS, version 22, IBM) software package. A Pillai’s mixed MANOVA (2 × 2 factorial design) was carried out with gender (men, women) and treatment (before, after) as independent variables and with RSES, THS, BDI, BAI, and HS as dependent variables. The effect sizes (Cohen’s *d*) were calculated and interpreted according to Cohen (1988): 0.20 as a small effect, 0.50 as a medium-sized effect, and 0.80 or over as a large effect. The significance level was set at 5%.

### Ethical considerations

The study complied with the Declaration of Helsinki.^25^ Strict compliance with the ethical requirements laid down in the treatment contract was ensured. These requirements, namely 1) the individual protection requirement, 2) the information requirement, 3) the consent requirement, and 4) the confidentiality requirement, were designed to protect the participants from any physical and/or psychological harm, violation or humiliation, and to protect their integrity against improper disclosure.^26^ The patients were informed that their participation in the study was voluntary and that they could withdraw from it at any time without consequence. All patients received written and verbal information on the purpose of the data collection and were informed that the results obtained would be published in the form of a scientific article. The participants were ensured that their individual answers would be anonymized in the results and that all data would be managed confidentially. Written consent was obtained from all participants.

## Results

### Characteristics of the study population

Of the 50 clients who took contact with the clinic during the study period, 35 individuals (18 male, 17 female) completed the four-month intensive treatment program. The participants’ average age was 45.6 years (SD = 11.79, range = 24–65). Fourteen participants were in a relationship and 21 were single. Twenty-four participants were parents and 11 had no children. All of the participants had previous healthcare system and/or social services records. Before admission to treatment each participant underwent clinical screening establishing their substance use and dependence.

### Effect of integrated intensive CBT treatment

The analysis showed significant effects for treatment (*p* < 0.001, *Eta*^2^ = 0.77, power > 0.99), and for the interaction of treatment × gender (*p* = 0.005, *Eta*^2^ = 0.50, power = 0.94), but not for gender by itself (*p* = 0.24, *Eta*^2^ = 0.27, power = 0.49). Univariate *F* tests showed significant treatment effects for all the dependent variables: RSES [*F* (1, 33) = 57.13, *p* < 0.001]; THS [*F* (1, 33) = 37.07, *p* < 0.001]; BDI [*F* (1, 33) = 50.34, *p* < 0.001]; BAI [*F* (1, 33) = 42.77, *p* < 0.001]; and HS [*F* (1, 33) = 9.85, *p* = 0.004]. As regards the interaction effect of treatment × gender the univariate *F* tests showed no significant interactions.

### Changes during integrated intensive treatment by gender

At the pre-treatment assessment, there were no significant differences measured between male and female patients’ mental health variables. The strongest but not significant difference was measured in the level of depression between male and female patients (BDI 20.39 for male patients and 26.35 for female patients, *p* = 0.15).

At the post-treatment assessment one significant difference was measured between the genders in the assessed mental health parameters, namely the Pathway THS subscale (*p* = 0.027), where female patients scored higher.

**Figure 1:**
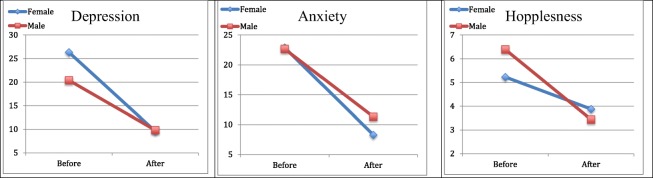
Changes in the scale of depression, anxiety and hopelessness during integrated intensive CBT treatment in male and female patients

**Figure 2:**
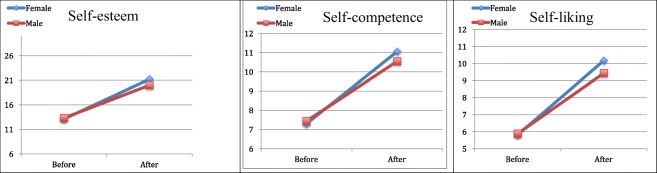
Changes in the scale of self-esteem scale and its dimensions, self-competence and self-liking, during integrated intensive CBT treatment in male and female patients

**Figure 3:**
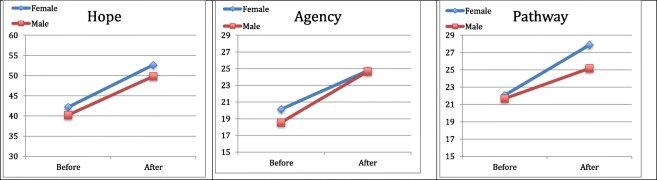
Changes in the scale of hope as well as its subscales agency and pathway, during integrated intensive CBT treatment in male and female patients

Table 1 summarizes the mean scores reported by the patients pre- and post-treatment. The changes in all variables were significant and had a large effect size (Cohen’s *d* from 0.67 to 1.51) in both genders, except in the variable hopelessness (HS) in female patients where the alleviation of hopelessness was not significant but had a medium effect size (Cohen’s *d* of 0.32). The strongest effects of the treatment could be measured in different areas in male and female patients. In female patients the integrated intensive CBT treatment resulted in significant and dramatic improvements in their scores pertaining to depression and anxiety (Fig. 1). In male patients the highest effect size could be measured within the self-esteem (self-liking) (Fig. 2) and the hope (agency) scales (Fig. 3).

**Table 1:** Descriptive pre- and post-treatment data and the level of change in the defined health variables in female and male patients separately

	Female (*n* = 17)					Male (*n* = 18)				
	BEFORE M (SD)	AFTER M (SD)	Change M (SD)	*p*	Cohen’s *d*	BEFORE M (SD)	AFTER M (SD)	Change M (SD)	*p*	Cohen’s *d*
Self-esteem (RSES) (0–30)	13.12 (5.42)	21.24 (6.37)	8.12 (6.59)	< .001	1.23	13.33 (7.11)	20.00 (5.06)	6.67 (4.90)	< .001	1.36
Self-competence (0–15)	7.29 (3.00)	11.06 (3.88)	3.76 (3.42)	< .001	1.10	7.44 (4.29)	10.56 (2.66)	3.11 (3.12)	.001	1.00
Self-liking (0–15)	5.82 (2.88)	10.18 (2.72)	4.35 (3.30)	< .001	1.32	5.89 (3.34)	9.44 (2.71)	3.56 (2.50)	< .001	1.42
Trait Hope Scale (THS) (8–64)	42.18 (11.34)	52.65 (8.819)	10.47 (11.53)	.002	0.91	40.22 (10.21)	49.83 (7.28)	9.61 (7.71)	< .001	1.25
Agency (4–32)	20.12 (7.07)	24.76 (4.63)	4.65 (6.98)	.014	0.67	18.56 (6.14)	24.67 (3.85)	6.11 (4.70)	< .001	1.30
Pathway (4–32)	22.06 (5.02)	27.88 (5.01)	5.82 (5.57)	.001	1.05	21.67 (4.80)	25.17 (4.27)	3.50 (4.45)	.004	0.79
Beck Depression Inventory (BDI) (0–63) ^a^	26.35 (10.29)	9.59 (7.98)	−16.76 (11.12)	< .001	1.51	20.39 (15.76)	9.83 (12.09)	−10.56 (11.63)	.001	0.91
Beck Anxiety Inventory (BAI) (0–63) ^a^	22.88 (9.75)	8.29 (5.61)	−14.59 (10.11)	< .001	1.44	22.67 (14.58)	11.33 (10.30)	−11.33 (13.06)	.002	0.87
Hopelessness Scale (HS) (0–20) ^a^	5.24 (2.97)	3.88 (3.59)	−1.35 (4.18)	.201	0.32	6.39 (5.23)	3.44 (3.38)	−2.94 (3.92)	.005	0.75

^a^Negative means change value indicates improvement in the assessed attribute

## Discussion

This research project had two objectives: (a) to evaluate an integrated intensive CBT group treatment for individuals with substance-related syndrome in an outpatient treatment program, and (b) to study eventual gender differences of the outcome.

For the evaluation of this specific treatment program we chose psychological measures that asses the patient’s level of vulnerability (i.e., anxiety [BAI], depression [BDI] and feelings of hopelessness [HS]) and strength of mind (i.e., self-esteem [RSES] and hope [THS]). It is shown that motivation factors to remain free from use of substances include increased self-esteem and the experience of meaningfulness and hope,^27^ while anxiety, depression, and even the feeling of hopelessness are risk factors for substance use.^10, 11, 27, 29, 30^ Therefore, any significant decrease in substance use risk factors and increase in protective factors indicate the success of the treatment program.

We can conclude that this integrated intensive CBT treatment had significant and strong (high effect size) positive effects for patients with substance-related syndrome, which supports the findings of previous research.^11, 13, 28^

Two of the common coexisting psychiatric conditions with substance-related syndrome are depression and anxiety.^29, 30^ This study showed that four months of integrated intensive CBT group treatment has the capacity to improve, with a high effect size, the affective symptoms in all patients. It is well known that affective symptoms, experience of depression and anxiety disorder often lead to the use of different substances.^10, 11, 28, 30, 31^ Through substance use, the individual attempts to improve his or her mood and to reduce depression and anxiety symptoms. The habit of using different substances to improve one’s everyday mood is so common, an occurrence that there is even a name for the phenomenon: self-medication. The term simply suggests that a person is trying to get relief from a problem that is causing him or her suffering. That the study could measure significant reductions in the experiences of anxiety, depression, and hopelessness, after four months of integrated intensive CBT treatment, is of importance because reductions of a person’s depression and anxiety symptoms serve as protective factors against relapse in substance use^28, 32^ and the reduction in hopelessness is important because hopelessness often coincides with depression, self-harm, and/or suicidal behavior.^18^ The positive impact of this integrated intensive CBT treatment is proven to be high, as it could significantly reduce psychological risk factors that may lead to substance use.

The other variables used in this study to evaluate the change of the mental health of patients with substance-related syndrome as a result of the given treatment were self-esteem, hope, and hopelessness. Self-esteem is of vital importance for the experience of being able to manage life challenges.^33^ Global self-esteem can be divided into two sub categories: self-competence and self-liking. Self-competence is about the individual being able to rely on his or her own ability to achieve a desired outcome by using his or her will.^22^ Self-liking is the individual’s experience of whether he or she is a good or bad person in a social context.^22^ In this study, it was found that global self-esteem, as well as its two dimensions (self-competence and self-liking), improves significantly after integrated intensive CBT treatment. Previous studies have shown the importance of enhanced self-esteem for the prevention of relapse in addiction.^34^ Patients whose self-esteem (self-liking and self-competence) has been strengthened are better armed to meet the various challenges of life and have better coping strategies than before, which strengthen the positive impact of this type of treatment in the prevention of relapse.

Self-esteem—and emotions in general—is dependent on the individual’s feelings of hope.^23^ With a hopeful attitude, the individual can set new goals and is more capable of achieving the goals.^24^ As the person accomplishes new goals, his or her experience of hope and self-esteem increases. Having a sense of hope is also an indicator of mental well-being, which is an important component of positive psychology.^35^ In positive psychology, one focuses on the individual’s strengths and assets with the premise that they can create protections against and regulate the impacts of the stressful life events that inevitably occur in life.^36^

The concept of hope can also be divided into two dimensions: “agency” and “pathway”.^23^ Snyder and colleagues^23^ define “agency” as a belief in being able to translate one’s decisions into actions, and “pathway” as path-finding thinking. “Agency” has proved to be the only contributing factor in hope that is of practical importance.^24^ In addiction care, it is important to raise the patients’ hopeful attitude towards the future, as well as his or her self-esteem. By doing this, the therapist helps enhance the patient’s “agency,” thus directly increasing his or her self-esteem. These interacting components of self-esteem and self-competence, self-liking, hope, “agency,” and “pathway” reinforce each other.^24^

Increases in all of the health components mentioned earlier were measured in this study after completion of the integrated intensive CBT outpatient treatment. After undergoing this specific treatment, the patients with substance-related syndrome gained higher experience of hope, indicating an improvement of their problem-solving abilities, as well as enhanced faith in their own ability, indicating an improvement of their ability to handle future challenges and stresses that may lead to relapse.

As regards the study’s other aim, i.e., to identify gender-specific effects, it was surprising that there were no significant differences in the treatment’s effects on male and female patients. Addiction care has addressed the need for more knowledge about gender-specific effects in care and possible development of gender-specific treatments. This study showed that integrated intensive CBT treatment, during which male and female patients participate in mixed groups (groups that include male and female patients at the same time), results in significant improvements for both genders.

Previous studies and reports have shown that there is a higher prevalence of coexisting psychiatric illnesses, which are also more severe, among females with substance use and dependence.^1, 3, 37^ Indeed, female patients in this study reported more depression and anxiety symptoms than male patients at the time of admission; however, these differences were not significant. Also, while female patients’ feeling of hopelessness decreased, it was the only measure that did not improve significantly during the four-month treatment period. Consequently, future studies need to investigate whether or not gender-specific treatment may affect risk factors of substance use in females.

An interesting observation in this study was that the female patients were younger (but not significantly so) than the male patients, which was unexpected because previous surveys have shown that women generally seek help later in life.^1^ It is also noteworthy that, contrary to the findings of the latest national and international investigations, which show that about three times more men than women seek and receive treatment for substance-related problems,^2, 3^ this study had an almost equal gender distribution (18 male and 17 female patients). Both the number and younger age of the female patients in the treatment program can be a local phenomenon or a new and very positive care-seeking trend among women.

## Strengths and Limitations

A limitation of this study was the modest size of the study population, which has an effect on the generalizability of the results. This limitation was due to time and clinic capacity constraints. The clinic in question is one of few, possibly the only, clinic providing the studied form of addiction treatment in Sweden, which limited the potential number of participants. This is the first published study in Sweden that evaluates a CBT-based integrated intensive addiction care treatment. The absence of previous studies of this kind may be due to a lack of resources to gather evaluation data in similar clinical settings. Two strengths of this study were that the clinical setting provided good opportunities to observe the patients in the course of the clinic’s everyday operations during the four-month treatment period without having to modify the settings or construct artificial experiments, and the even gender distribution of the study’s population.

## Implications for Behavioral Health

The study results prove that all outcome measures of mental health improved in patients with substance-related syndrome, which indicates that integrated intensive CBT treatment entails positive effects in both genders. The study’s results emphasize the effectiveness and value of implementing integrated treatment programs, instead of sequential or parallel treatment programs, for patients with substance-related syndrome.

Further research on the benefits of the integrated intensive form of treatment in outpatient addiction care is needed and should be completed with measures of substance dependence, such as craving and relapse rate.
